# Population pharmacokinetic model library for isavuconazole: development and quality control

**DOI:** 10.3389/fmicb.2026.1930025

**Published:** 2026-10-06

**Authors:** Qing Tang, Yuhua Zhao, Jianping Zhu, Jieqiong Liu, Gang Liang, Yi Yang, Renai Xu, Zhenwei Yu

**Affiliations:** 1 Department of Pharmacy, Sir Run Run Shaw Hospital, School of Medicine, Zhejiang University, Hangzhou, China; 2 Research Center for Clinical Pharmacy, College of Pharmaceutical Science, Zhejiang University, Hangzhou, China; 3 Department of Pharmacy, Affiliated Xiaoshan Hospital, Hangzhou Normal University, Hangzhou, China; 4 Department of Pharmacy, First Affiliated Hospital of Wenzhou Medical University, Wenzhou, China

**Keywords:** clearance, covariate, isavuconazole, model library, population pharmacokinetics

## Abstract

**Background and objective:**

Isavuconazole is the first-line option for the treatment of invasive aspergillosis and mucormycosis. However, it has large pharmacokinetic (PK) variabilities, and suboptimal exposure is concerned. This study aimed to develop an isavuconazole population pharmacokinetic (PPK) library that contributes to model-informed precision dosing (MIPD) and dose optimization.

**Methods:**

Following a systematic literature search and study inclusion, a library of isavuconazole PPK models was developed. The model characteristics and parameters estimated were extracted and compared. The AUC distributions at steady status across different models were compared, and the effects of covariates on PK parameters were analyzed. The model library was used to estimate target achievement with the PK/PD target of AUC/MIC 33 for Aspergillus through Monte Carlo simulations.

**Results:**

A total of 955 unduplicated records were initially identified from PubMed, Web of Science, and Embase. After screening and eligibility assessment, 14 studies were ultimately included in the model library. The quality scores of the included studies ranged from 35 to 45. The included PPK models had high reporting quality and covered a diversity of patients, with the two-compartment model being the most common. The total clearance of isavuconazole ranged from 1.33–5.34 L/h. Body weight, body surface area (BSA), body mass index (BMI), albumin levels, and liver function markers were the most common significant covariates, and their overall impact met the clinically significant criteria. The simulation results indicate that for a susceptive threshold of MIC 1 mg/L, most models (11/14) achieved PTA over 90%, while dose optimization may be required for critically ill patients, hematological patients and patients with abnormal covariates.

**Conclusion:**

This study successfully developed a PPK library for isavuconazole, which would support the precision dosing of isavuconazole.

## Introduction

Isavuconazole is a newer-generation broad-spectrum triazole antifungal administered as the water-soluble prodrug isavuconazonium sulfate and has become an important first-line therapeutic option for invasive aspergillosis (IA) and a well-established treatment for mucormycosis (Shirley and Scott, 2016; Seyedmousavi et al., 2015; Jenks et al., 2018; Chen et al., 2023). It exhibits potent activity against clinically important Aspergillus species, including isolates resistant to amphotericin B, caspofungin, or itraconazole (Pfaller et al., 2023). Like other triazole antifungals, isavuconazole exerts its antifungal effect by inhibiting cytochrome P450-dependent lanosterol 14α-demethylase, an enzyme involved in the biosynthesis of ergosterol, which is a component of fungal cell membranes (Livermore and Hope, 2012; Administration USFaD, 2025). In contrast, the mammalian sterol biosynthetic pathway is considerably less susceptible to azole-mediated inhibition, contributing to the favorable therapeutic selectivity of this drug class (Ghannoum and Rice, 1999). The efficacy of isavuconazole has been established in the SECURE trial, which demonstrated noninferiority to voriconazole with respect to the primary treatment outcome in patients with invasive mold diseases (Maertens et al., 2016).

Previous studies in healthy volunteers have shown that isavuconazole exhibits a large apparent volume of distribution (V) ranging from 308 to 542 L and a mean plasma half-life of 130 h (Administration USFaD, 2025; Desai et al., 2016a). Isavuconazole is highly protein bound (>99%), with the majority of circulating drugs bound to albumin (Administration USFaD, 2025; European Medicines Agency, 2024). Both *in vitro* and *in vivo* studies have demonstrated that isavuconazole is primarily metabolized by the cytochrome P450 enzymes CYP3A4 and CYP3A5, with minor contributions from UGT-mediated biotransformation pathways, which consequently raises concerns about drug–drug interactions (Administration USFaD, 2025; European Medicines Agency, 2024; Huang et al., 2023; Sang et al., 2025).

Preclinical and clinical investigations have identified the ratio of the area under the concentration–time curve to the minimum inhibitory concentration (AUC/MIC) as the principal pharmacokinetic/pharmacodynamic (PK/PD) index associated with the antifungal activity of isavuconazole (Pfaller et al., 2023; Kovanda et al., 2017; Verweij et al., 2009). The currently approved dosing regimen generally achieves adequate PK/PD target attainment against susceptible fungal pathogens. Nevertheless, substantial interindividual variability in drug exposure has been reported in specific patient populations (Bolcato et al., 2022). Moreover, the clinical relevance of defined exposure thresholds and the role of routine therapeutic drug monitoring (TDM) remain subjects of ongoing debate (Desai et al., 2017; Tan et al., 2025). Hence, personalized dosage recommendations and tailored adjustments, usually informed by measured observations and model-predicted exposure, are crucial to optimize treatment outcomes.

PPK models provide a quantitative framework for characterizing the pharmacokinetic properties of isavuconazole and for evaluating the contribution of patient-specific covariates to pharmacokinetic variability (Minichmayr et al., 2024). Over the past decade, numerous PPK models of isavuconazole have been developed across diverse patient populations, including critically ill patients in intensive care units (ICUs), solid-organ and hematopoietic stem-cell transplant recipients, patients with hepatic impairment, and pediatric populations (Chen et al., 2023; Wu et al., 2020; Peña-Lorenzo et al., 2025). Furthermore, when integrated with Bayesian forecasting approaches, PPK models can combine individual patient characteristics with therapeutic drug monitoring data to support model-informed precision dosing (MIPD), thereby facilitating the optimization of individualized treatment strategies (Minichmayr et al., 2024). Despite the growing number of published PPK studies on isavuconazole, existing models have not yet been systematically integrated, reconstructed, or quantitatively compared within a unified analytical framework. Developing a comprehensive library of isavuconazole PPK models would therefore not only facilitate objective model evaluation and advance quantitative pharmacological research but also promote the broader implementation of MIPD strategies in clinical practice. Thus this study aimed to (1) assess the quality of published isavuconazole PPK models and develop a quality-controlled model library; (2) characterize between-model variability in drug exposure and evaluate the clinical relevance of covariate effects; and (3) assess the adequacy of standard dosing regimens and PTA using the model library.

## Materials and methods

### Search strategy

Three databases, PubMed, Embase, and Web of Science, were systematically searched for studies reporting PPK models of isavuconazole published before December 2025, in accordance with the Preferred Reporting Items for Systematic Reviews and Meta-Analyses (PRISMA) guidelines (Page et al., 2020). The search strategy focused on drug-related keywords such as “isavuconazole,” “Cresemba,” or “BAL 4815” combined with terms specifically indicating PPK modeling approaches, including population pharmacokinetic, NONMEM, and MONOLIX.

### Inclusion and exclusion criteria

This study focusing on population pharmacokinetics for isavuconazole could be included if it met the following inclusion criteria: (1) the study was performed in humans, including healthy people and patients; (2) population pharmacokinetic analysis was carried out, and key parameters were reported; and (3) the study was published in English. The articles were excluded if they were (1) *in vitro* or animal studies; (2) conventional pharmacokinetic studies or bioequivalence studies; or (3) pure PPK simulation studies.

### Literature quality evaluation

For the evaluation of the included PPK studies, a 46-item checklist adapted from guidelines and previously reported literature was used (Supplementary Table S1) (Kanji et al., 2015; Xu et al., 2024). The assessment was divided into five domains: title and abstract, introduction, methods, results, and discussion and conclusion. Each adequately reported item was awarded 1 point, whereas incompletely reported items were assigned 0.5 points.

### Data extraction

The relevant information was extracted from the included studies, and the main items included the following aspects: (1) study design features: year of publication, authors, country, study type (prospective/retrospective), number of subjects and observations, dosing regimen, sampling schedule, and quantification method; (2) participant demographics: sex, age, and weight; (3) PPK model characteristics: structural model, formula and parameters of the final model, software used, model evaluation method, covariate analysis method, covariates screened and incorporated, interindividual variability, and residual unexplained variability; (4) simulation target (if applicable); and (5) model application.

### Quality control of the model library

To characterize the between-study variability of pharmacokinetic parameters and evaluate the consistency of models included in the library, we reimplemented the models by encoding the models in NONMEM (version 7.6.0) and performed Monte Carlo simulations. For studies that did not publicly provide NONMEM control files, we reconstructed the models based on the structural models and parameter estimates reported in the literature. When necessary, simplified compartmental structures were applied to facilitate model reconstruction while preserving the key pharmacokinetic parameters and characteristics reported in the original studies. The reconstructed models were further evaluated by comparing the estimated key PK parameters and simulated concentration–time profiles with those reported in the original publications. And the specific simulation procedure was as follows: A total of 1,000 virtual patients were simulated with 200 mg isavuconazole administered either i.v. or orally once daily according to the study. To avoid bias caused by differences in absorption processes, the absorption parameters (Ka, ALAG, F) that existed only in the oral model were included solely in the analysis of the oral model. The covariates were set to typical values for each study to eliminate the influence of covariates. We incorporated the interindividual variability reported in various literature sources. After steady state was achieved, the corresponding AUC was calculated using the equation AUC=Dose/CL(CL/F) or through numerical integration by simulating the concentration-time curve. Boxplots of AUCs across studies were generated using the “ggplot2” package (version 4.0.1) in R software.

The effects of variates on clearance and distribution parameters, two important pharmacokinetic parameters associated with AUC and C_trough_, were analyzed. The clearance parameters were mainly the clearance rate (CL) of the central department. Distribution-related parameters included V in the one-compartment model, central volume of distribution (V_c_/V_1_), peripheral volume of distribution (V_p_/V_2_), and second peripheral volume (V_3_) in the multiple-compartment models. For continuous covariates, the median value of the original study was used as the median, and the range of values across studies was used as the endpoint. For binary covariates, the baseline category was used as the reference group, and another category was used as the comparison group. To facilitate comparisons across different studies, the covariate effects reported in each model were uniformly converted into relative change rates of parameter estimates compared to the reference group and details in Equation 1. Then, forest plots were generated using the “ggplot2” package (version 4.0.1) in R to visualize the effects of the identified covariates. Changes within the 80%–125% range were considered clinically insignificant.

$$\begin{array}{l} \text{Covariate Effect}(\%)\\ =\frac{\text{Parameter Value}\;\mathrm{at}\;\text{Upper or Lower Limit}}{\text{Parameter Value}\;\mathrm{at}\;\text{Reference Value}}\times 100 \end{array}$$ (1)

### Application of the PPK model library

The PPK model library was applied to assess the dose suitability among different models or populations. Every 5,000 virtual subjects were simulated for each model/population. The maintenance dose was fixed to 200 mg every 24 h by oral or intravenous infusion. The covariates were set to the median value of each model. For the nonlinear protein binding model, individual steady-state total drug exposure was computed using Box–Cox transformation parameters and protein binding kinetic parameters (Jansen et al., 2023). The steady-state 24-h area under the curve (AUC_ss_) was calculated as the ratio of dose to individual clearance, and the AUC/MIC ratio was evaluated. The PK/PD target for *Aspergillus* was defined as an AUC/MIC ratio of 33 based on previous PK/PD analyses (Bergmann et al., 2024). The MIC was set to EUCAST susceptibility breakpoints of ≤1 mg/L. (Arendrup et al., 2016) PTA is characterized as the proportion of virtual subjects who achieve the preset PK/PD targets, expressed as a percentage.

## Results

### Literature search and study inclusion

A total of 955 unduplicated records were initially identified from the PubMed, Web of Science and Embase databases. Finally, 14 studies were included in our model library for subsequent analysis. The PRISMA flowchart for study inclusion is presented in Figure 1.

**Figure 1:**
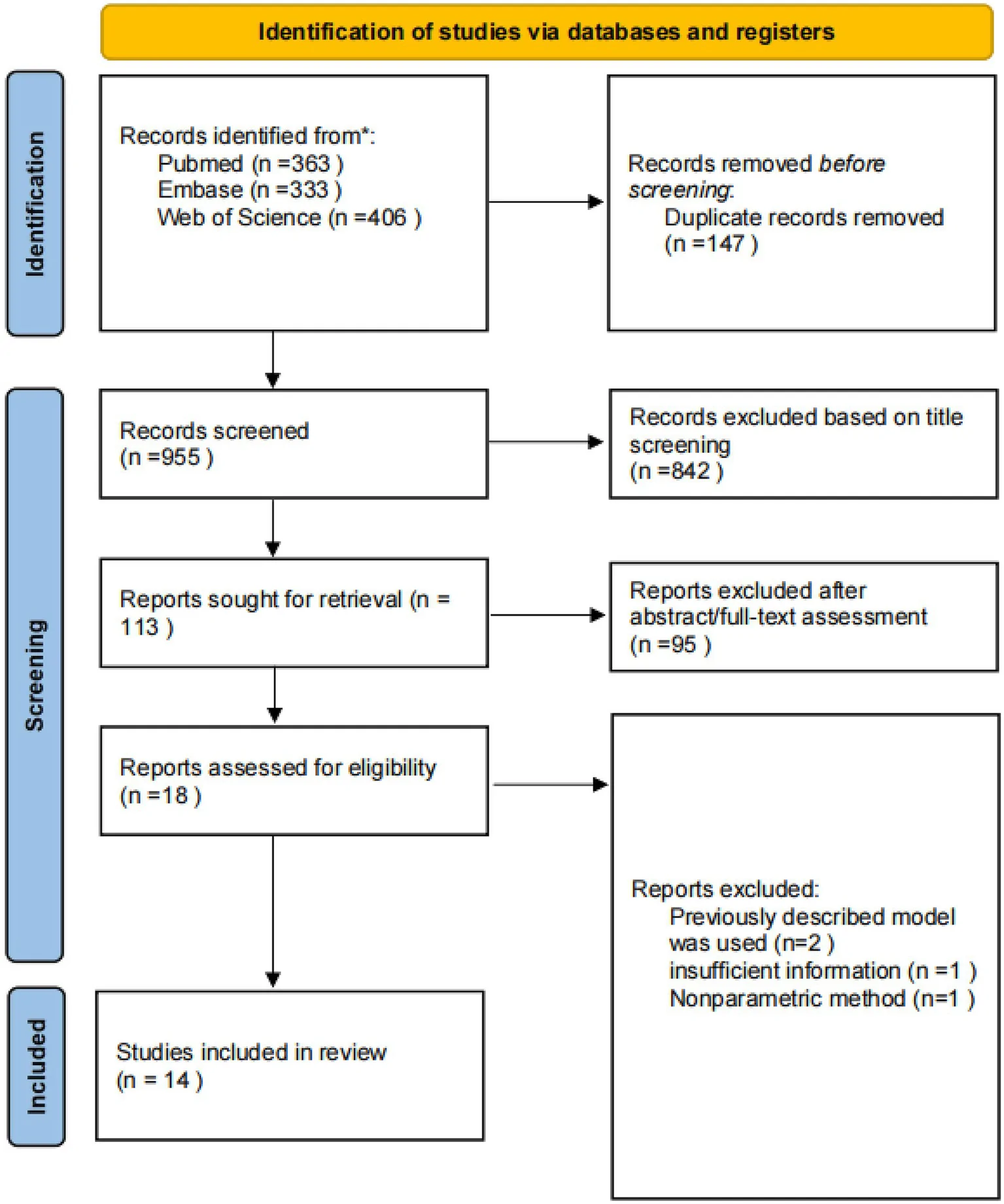
PRISMA flow diagram for the selection process of isavuconazole population pharmacokinetics (PPK) studies in the library.

### Evaluation of literature quality

The 14 included population pharmacokinetic studies were evaluated using a quality assessment checklist. The quality scores of the included studies ranged from 35 to 45 points, corresponding to compliance rates of 76.1% to 97.8%. With the exception of one study, all others scored above 38 points (Peña-Lorenzo et al., 2025). Key elements such as the title, abstract, study objectives, population pharmacokinetic modeling methods, parameter estimation, and model evaluation were reported relatively comprehensively. None of the studies included external validation. Detailed quality assessment results are presented in (Supplementary Table S2).

### Study and PPK model characteristics

The key characteristics of these selected studies are summarized in Table 1. These studies were published between 2016 and 2025 and included six prospective studies and eight retrospective studies. The sample sizes ranged from 18 to 421 participants. Three studies included both patients and healthy volunteers (Desai et al., 2016a,b; Desai et al., 2019), whereas the remaining studies enrolled only patients with invasive fungal infections (Kovanda et al., 2017; Wu et al., 2020; Peña-Lorenzo et al., 2025; Jansen et al., 2023; Shirae et al., 2023; Perez et al., 2023; Arrieta et al., 2021; Martín-Cerezuela et al., 2024; Kovanda et al., 2016; Rabault et al., 2025; Cojutti et al., 2021). Only one study recruited a pediatric population aged 1 to 18 years, while all other studies included adults only (Arrieta et al., 2021). Among these studies, four focused on critically ill patients (Jansen et al., 2023; Perez et al., 2023; Martín-Cerezuela et al., 2024; Rabault et al., 2025), and one specifically enrolled solid organ transplant (SOT) recipients (Wu et al., 2020). Supplementary Table S3 shows the experimental designs and dosing regimens of the included studies. These studies used various quantification methods to determine isavuconazole concentrations, and more than half of the studies used both oral and intravenous administration routes.

**Table 1:** Characteristics of included population pharmacokinetics studies.

ID	Author (Publication year)	Country (Type of study)	Patient group	Number of subjects	Number of observations	Gender (male/female)	Age (year) Mean ± SD Median (Range)	Weight (kg) Mean ± SD Median [Range]
1	Kovanda et al. (2016)	UK, USA (Prospective)	Renally impaired IA patients; patients with invasive fungal disease (IFD) caused by emerging molds, yeasts, and dimorphic fungi	136(131/7)	458	NR	NR	70.2 ± 19.5
2	Desai et al. (2016a)	USA, Switzerland (Retrospective)	Healthy subjects, subjects with mild hepatic impairment, and subjects with moderate impairment	32(Healthy), 32(With mild hepatic impairment), 32(With moderate hepatic impairment)	2016	Healthy (21/11); With mild hepatic impairment (21/11); With moderate hepatic impairment (21/11)	Healthy: 50(40–64); With mild hepatic impairment:54(37–64); With moderate hepatic impairment:54(42–64)	Healthy:78(53–107); With mild hepatic impairment:76(56–105); With moderate hepatic impairment:76(58–103)
3	Desai et al. (2016b)	USA (Retrospective)	Healthy subjects and patients with IFIs	421: 189 (healthy), 232 (patients)	6,363 (5828- healthy people,535 -patients)	Healthy (140/49) Patients with IFIs (132/100)	Healthy: 43.0(19.0–85.0); Patients with IFIs: 54.0(17.0–82.0)	Healthy subjects:77.8(51.7–118.3); Patients with IFIs:67.0(41.0–127.7)
4	Kovanda et al. (2017)	Multicenter international (Prospective)	Patients with IFD, including invasive aspergillosis (IA), mucormycosis, and other rare fungal infections; subgroup analysis in patients with and without mucositis	250: 56(with mucositis), 194(without mucositis)	NR	155/95	50.3 ± 16.1 52(18–92)	70.0 ± 18.3
5	Desai et al. (2019)	USA, Japan (Prospective)	Healthy nonelderly (18–45 years) and elderly (≥65 years) males and females	48	882	12/12(nonelderly) 12/12(elderly)	Nonelderly male: 30.3 ± 7.59(19–42); Nonelderly female: 29.5 ± 6.45 (22–45); Elderly male: 70.9 ± 5.74 (65–85); Elderly female: 71.5 ± 5.50 (66–84)	Nonelderly male: 80.17 ± 12.90(57.0–105.7); Nonelderly female: 70.01 ± 10.49(56.3–86.5); Elderly male: 77.88 ± 11.58(59.1–94.1); Elderly female: 61.03 ± 8.71(51.7–80.0)
6	Wu et al. (2020)	USA, China (Retrospective)	In solid-organ transplant recipient	79	471	49/30	53.9 ± 14.4	71.6 ± 19.4
7	Arrieta et al. (2021)	USA, Switzerland (Retrospective)	Immunocompromised pediatric patients	46	45	20/7(i.v. cohort) 9/10(oral cohort)	i.v. cohort: 10.0(1–17) oral cohort: 12.0(6–17)	i.v. cohort: 33.7(10.9–103.5) oral cohort: 42.6(18.3–92.8)
8	Cojutti et al. (2021)	Italy (Retrospective)	Patients with IFIs, non-Aspergillus IFD	50	199	31/19	61.5(51.3–72)	65(55.5–71.5)
9	Jansen et al. (2023)	Netherlands, Belgium (Prospective)	In critically ill patients	19	152 (unboundand); 152 (total isavuconazole concentrations)	14/5	67 (41–73)	80 (45–130)
10	Shirae et al. (2023)	Japan (Retrospective)	Patients with deep-seated mycoses	196	4,421 (4084- healthy subjects;337- patients)	56/13(Patient) Healthy: 100%male	29.0 (20–42) 26.5 (20–44) 68.0 (24–87)	59.5 (51.5–72.0) 61.0 (50.8–79.4) 50.6 (31.0–81.4)
11	Perez et al. (2023)	France (Retrospective)	In critical care patients with COVID-19-associated pulmonary aspergillosis	18	65	14/4	65 (IQR56-70)	85 (IQR79-92) 29.2 (25.6–31.8)
12	Martín-Cerezuela et al. (2024)	Spain (Prospective)	In critically ill patients	24	71	17/7	53.2 ± 16.4	75.1 ± 12.6
13	Peña-Lorenzo et al. (2025)	Spain (Prospective)	In hematologic patients	52	121	23/29	59 (19–89)	70 (45–150)
14	Rabault et al. (2025)	France (Retrospective)	Critically ill patients treated for mold infections (aspergillosis, CAPA, IAPA, mucormycosis)	22	134	17/5	58(IQR55-61)	90(77–101.5)

IA, invasive aspergillosis; IFD, invasive fungal disease; USA, United States of America; IFIs, invasive fungal infections; IQR, interquartile range; IPA, invasive pulmonary aspergilosis; NR, not record.

The modeling strategies and final PK parameter estimates of the included studies are summarized in Table 2. Among these studies, instead of using NONMEM for model development, three used Pmetrics, and two studies used Monolix (Kovanda et al., 2017; Perez et al., 2023; Kovanda et al., 2016; Rabault et al., 2025; Cojutti et al., 2021). More than half of the studies applied a two-compartment model to describe the pharmacokinetics of isavuconazole, whereas three studies used a one-compartment model, likely because sparse sampling schedules could not adequately characterize the distribution phase of a two-compartment model (Peña-Lorenzo et al., 2025; Perez et al., 2023; Martín-Cerezuela et al., 2024). In contrast, the study by Arrieta AC et al. combined pediatric PK data from multiple administration routes with adult intravenous PK data, and the richer dataset provided sufficient information to support the development of a linear three-compartment model with first-order elimination (Arrieta et al., 2021).

**Table 2:** Modeling strategies and final pharmacokinetics parameters of included studies.

ID	Author	Software	Compartments	Population typical value	Inter-individual variability (IIV)	Residual unexplained variability	Model evaluation method	Simulation target	Modeling application
1	Kovanda et al. (2016)	Pmetrics (v1.3.2)	2-CMT	CL = 2.5 ± 1.6 L/h V_1=_ 361.2 ± 166.3 L	CL = 63%	NR	GOF(Observed vs. predicted); Bayesian posterior predicted; coefficient of determination [r (2)]; bias and precison	AUC/MIC Aspergillus spp.50.5 *C. albicans* 270/5,053 non-albicans Candida spp. 312	PTA analysis; evaluate PK characteristics; support clinical dosing regimen adequacy
2	Desai et al. (2016a)	NONMEM (version 7.2)	2-CMT	CL =2.540 L/h(healthy) = 1.550 L/h(mild) = 1.326 L/h(moderate) V_1=_ 51.4 L V_2=_ 41.0 L Q = 33.678 L/h(healthy) = 38.80 L/h(mild) = 63.554 L/h(moderate)	CL = 43.47% V_1_ = 21.23% V_2_ = 27.05% Q = 36.46%	Ln transformed add. Error = 17.88	GOF Bootstrap NPDEs pcVPC	C_trough_	Evaluate covariate effects
3	Desai et al. (2016b)	NONMEM (version 7.2)	2-CMT	CL =2.360 L/h (predominantly Caucasian) = 1.51 L/h(Asian) V_1=_ 49.1 L V_2=_ 417.0 L Q = 26.6 L/h	CL =31.3% (healthy subjects) = 62.44% (patients) V_2_ = 31.78% Q = 49.09%	prop. Err = 44.94%	GOF Bootstrap NPDEs	AUC/MIC (Aspergillus spp.) EUCAST EI50/80/90: 24.7/29.8/33.3 CLSI EI50/80/90: 50.4/55.7/59.0	Evaluate covariate effects; PTA analysis; dosing optimization
4	Kovanda et al. (2017)	Pmetrics (v1.4.1)	2-CMT	**Mucositis group:** K_a_ = 7.0 h^−1^ CL = 2.2 L/h V = 331.4 L *F* = 86.0% **Nonmucositis group:** K_a_ = 6.5 h^−1^ CL = 2.3 L/h V = 354.1 L *F* = 97.4%	Ka = 38–46% CL = 44–47% V = 47–52% *F* = 7–21%	Bias = 0.11; Imprecision = 0.938	GOF(Observed vs. median predicted plots); Bayesian posterior prediction; coefficient of determination (r^2^ = 0.813); bias and imprecision analysis	AUC_ave_ (Average area under the curve)	Assess whether oral isavuconazonium sulfate can be used in patients with mucositis; evaluate need for therapeutic drug monitoring or extended IV therapy
5	Desai et al. (2019)	NONMEM (version 7.2)	2-CMT	CL =1.99 L/h(males) = 2.13 L/h (nonelderly females) = 1.44 L/h (elderly females) V_1=_9.220 L V_2_ = 263 L Q = 22.2 L/h	NR	NR	GOF Bootstrap NPDEs	NR	Evaluate covariate effects; dose adjustment evaluation
6	Wu et al. (2020)	NONMEM (version 7.2)	2-CMT	CL = 4.28 L/h(female) = 2.73 L/h(male) V_1=_ 57.6 L V_2=_ 468 L Q = 37.4 L/h	CL = 48.7% V_2_ = 46.7%	prop. Err = 20.50%	GOF Bootstrap VPC NPDEs	AUC/MIC *C. albicans*5053 C. glabrata156 *A. fumigatus*50.5	Evaluate covariate effects
7	Arrieta et al. (2021)	NONMEM (version4.7.0)	3-CMT	CL = 2.55 L/h V_1_ = 17.8 L Q_3_ = 30.3 L/h V_2_ = 26.0 L Q_4_ = 24.5 L/h V_3_ = 254.0 L	CL = 45.16% V_2_ = 43.24% V_3_ = 68.11% Q_4_ = 45.71%	add. Err = 40.86	GOF Bootstrap	AUC_ss_ 60–233 mg·h/L	Evaluate pediatric PK; target attainment; dosing optimization
8	Cojutti et al. (2021)	Pmetrics	2-CMT	CL = 1.33 L/h V_1_ = 102.58 L V_2_ = 385.93 L Ka = 22.64 h^−1^ Q = 5.08 L/h	CL = 64.03% V_1_ = 47.35% V_2_ = 86.22% Q = 109.37% Ka = 15.66%	NR	GOF VPC NPDE	AUC_0-24h/_MIC *A. fumigatus* 33.4 *A. flavus* 33.4 C_trough_	Assessment of exposure variability and support for TDM in clinical practice
9	Jansen et al. (2023)	NONMEM (version7.5.1)	2-CMT	CL = 215 L/h Q = 2,590 L/h V_1_ = 2,330 L V_2 =_28200L B _max=_ 6.64 mg/L	CL = 107.8% B _max=_ 26.0%	Log transformed prop. Err Error _total_ = 10.2% Error _unbound_ = 14.3%	GOF pcVPC (SIR) MPE and NRMSE	AUC_ss_	Adaptive/individualized dosing based on total drug or albumin levels to achieve target unbound AUC.
10	Shirae et al. (2023)	NONMEM, (version7.4.3)	2-CMT	CL = 1.74 L/h V_2_ = 25.5 L V_3_ = 122 L Q = 19.2 L/h	CL = 31.6% Q = 20% V_3_ = 22.36%	prop. Err = 24%(Healthy) = 105% (Patient)	GOF pcVPC Bootstrap	AUC/MIC	PTA analysis; Exposure-safety evaluation (QTc prolongation and liver function)
11	Perez et al. (2023)	Monolix software (version 2021R2)	1-CMT	CL = 3.98 L/h V = 850 L	CL = 25.8% V = 39.9%	prop. Err = 17%	GOF NPDEs pcVPC Bootstrap	C_trough_	Dosing optimization
12	Martín-Cerezuela et al. (2024)	NONMEM (version5)	1-CMT	V = 122 L CL = 5.36 L/h	V = 67% CL = 45.3%	NR	GOF	NR	Exposure-response analysis(efficacy/safety); evaluation of CRRT and ECMO impact on PK.
13	Peña-Lorenzo et al. (2025)	NONMEM (version7.5.0)	1-CMT	CL = 3.83 L/h V = 226 L K_a_ = 22.6 h^−1^(Fixed)	CL = 20.61%	prop. Err = 7.16%	Bootstrap GOF pcVPC	Steady-state concentration (C_ss_) 2–5 mg/L	Evaluate covariate Effects; target attainment; dose optimization
14	Rabault et al. (2025)	Monolix software (version 2024R1)	2-CMT	CL = 5.4 L/h V_1_ = 46.7 L Q = 43.2 L/h V_2_ = 608.5 L	CL = 86% V_1_ = 112% V_2_ = 80%	NR	GOF VPC	AUC_0-24h/_MIC C_trough_	Dose optimization for critically ill ICU patients

*The parameters were estimated for unbound drug. Abbreviations: CMT, compartment; CL, clearance; Q, Q3 and Q4, inter-compartmental clearance values; V_1_, central volume of distribution; V_2_ and V_3_, peripheral volume of distribution; B_max_, maximum binding capacity; K_a_, first-order absorption rate constant; GOF, Goodness–of-fit Plots; pcVPC, prediction-corrected visual predictive check; SIR, Sampling Importance Resampling; MPE, mean prediction error; NRMSE, normalised root mean squared error; NPDE, normalized prediction distribution errors; VPC, visual predictive check; IWRES, individual weighted residuals; TDM, therapeutic drug monitoring; NONMEM, Nonlinear Mixed Effects Modeling; NR, no record.

Only one study did not include between-subject variability (BSV) (Desai et al., 2019). Across the remaining studies, the median BSV for clearance was 45.5% (range: 20.61%–107.8%). Residual unexplained variability (RUV) was mainly described using proportional error models or additive error models on the normal scale, with proportional residual variability ranging from 7.16 to 105% (Desai et al., 2016a; Wu et al., 2020; Shirae et al., 2023; Perez et al., 2023; Jansen et al., 2023; Peña-Lorenzo et al., 2025). A few studies used additive error models after ln transformation (additive error = 40.86) or proportional error models after log transformation (Desai et al., 2016b; Arrieta et al., 2021), while five studies did not clearly report the type of residual error model applied (Desai et al., 2019; Martín-Cerezuela et al., 2024; Kovanda et al., 2016; Rabault et al., 2025; Cojutti et al., 2021).

All included PPK models underwent internal validation. For basic diagnostic evaluation, all studies assessed model fit using goodness-of-fit plots (GOF). To further evaluate model stability and predictive performance, several advanced validation approaches were applied. Eight studies performed bootstrap resampling analyses, while five studies used prediction-corrected visual predictive checks (pcVPCs), particularly for datasets with complex dosing regimens or high concentration variability. Three used a standard visual predictive check (VPC), and five studies additionally incorporated normalized prediction distribution error (NPDE) analyses for statistical model diagnostics. In addition, observed vs. predicted plots, Bayesian posterior prediction, coefficient of determination, and bias and imprecision analysis were also used as criteria in two studies during covariate selection and model development. However, none of the included studies performed external validation.

Monte Carlo simulation was applied in 12 studies to evaluate pharmacokinetic target attainment, optimize dosing regimens, and assess covariate effects in special populations. Six studies used AUC/MIC-based PK/PD targets for PTA analysis against fungal pathogens. Two studies employed an AUC_ss_ to evaluate exposure attainment. Another two studies proposed adaptive or individualized dosing strategies based on total or albumin-adjusted unbound AUC exposure. Four studies used trough concentration (C_trough_) as the primary exposure target for dosing optimization. Most studies focused on covariate analysis and dose optimization, while several additionally evaluated PTA, target attainment, pediatric pharmacokinetics, individualized dosing strategies, and exposure–safety relationships.

Seven studies suggest that the standard isavuconazole dosing regimen provides adequate drug exposure in general patients and some special populations, without the need for routine dose adjustment (Wu et al., 2020; Peña-Lorenzo et al., 2025; Jansen et al., 2023; Desai et al., 2019; Perez et al., 2023; Arrieta et al., 2021; Kovanda et al., 2016). However, in specific populations, such as pediatric patients, individuals with high body weight, critically ill patients, infections caused by pathogens with high MIC values, and ICU patients, some studies support dose escalation when necessary to improve PTA and therapeutic outcomes. In these situations, TDM should be strengthened to reduce the risk of drug accumulation and toxicity.

### Quality control of the PPK library

#### Comparison of isavuconazole PK profiles

The AUC distributions simulated using the 14 PPK models are presented in Figure 2. As shown, the models developed by Cojutti et al. and Jansen et al. yielded higher AUC values than those reported in the other studies under the same dosing regimen, which may be attributable to their relatively lower estimates of CL and V (Jansen et al., 2023; Cojutti et al., 2021). In contrast, the last three studies exhibited comparatively lower systemic exposure.

**Figure 2:**
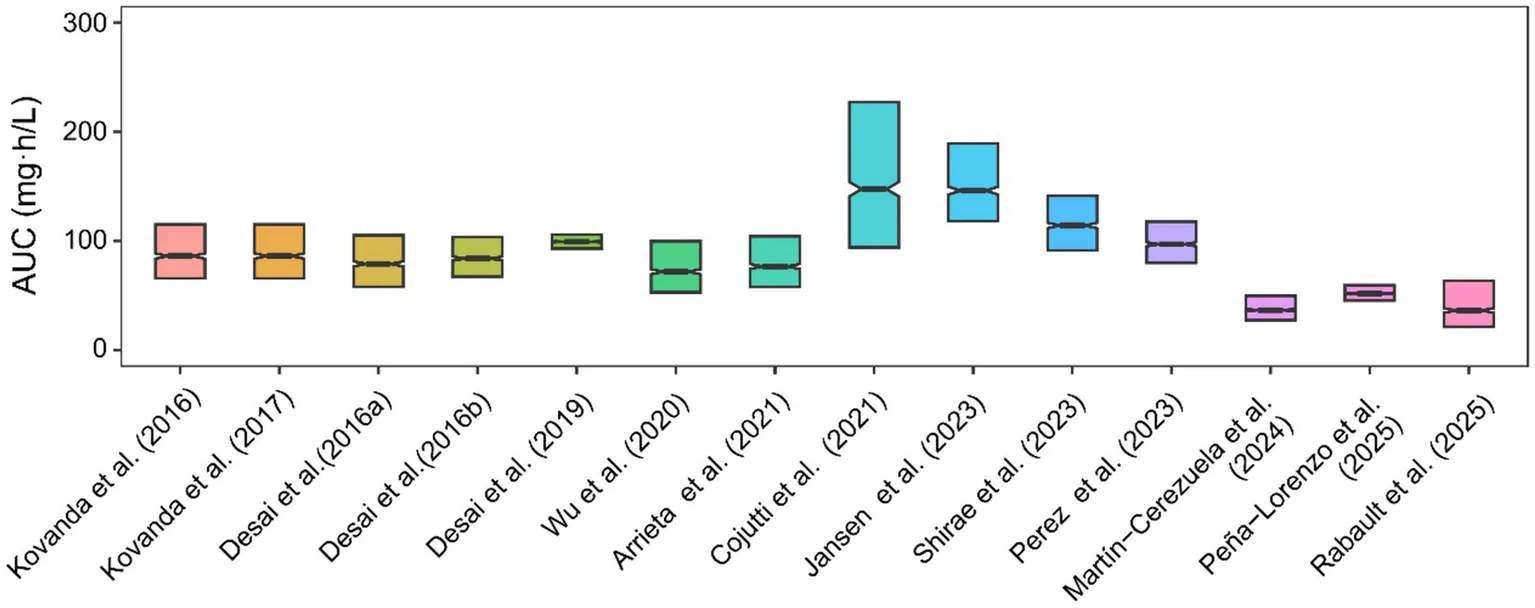
Isavuconazole main pharmacokinetic parameter (AUC) at steady state of patients. All patients received a loading dose of 200 mg ISA q8h for six doses, followed by a maintenance dose of 200 mg q24h either p.o. or i.v.

#### Covariate effect analysis

All covariates were examined for their impact on clearance-related and volume-related parameters. The details are presented in Table 3. Most studies employed forward inclusion and backward elimination methods for covariate selection. Seven studies did not identify any statistically significant covariates in the final model. Body size-related parameters were the most frequently included covariates in the final models. Three studies found a significant association between body mass index (BMI) and V_p_ (Desai et al., 2016a,b; Shirae et al., 2023), while another study reported that body surface area (BSA) significantly influenced CL (Peña-Lorenzo et al., 2025). Additionally, one study included lean body mass (LBM) in the CL model. Organ function-related indicators were also identified as important covariates in some studies (Shirae et al., 2023). Different studies have demonstrated significant effects of liver function markers, albumin (ALB) levels, and creatinine clearance (CLCR) on CL (Peña-Lorenzo et al., 2025; Desai et al., 2016b; Shirae et al., 2023). The influence of demographic factors on pharmacokinetic parameters varied across studies. For CL, race and sex were included in the final model either as separate or combined covariates. Disease status (COND) was retained in the final model in one study (Shirae et al., 2023). Regarding treatment-related factors, only one study evaluated the effect of renal replacement therapy (RRT) on CL (Perez et al., 2023).

**Table 3:** Investigated and identified covariates in population pharmacokinetic models of included studies.

ID	Author	Covariate analysis method	Covariates screened	Covariates incorporated	Formulation
1	Kovanda et al. (2016)	Graphical evaluation and linear regression analysis; covariate inclusion into structural model and likelihood comparison	Weight, BMI, Race, eGFR	NA	NR
2	Desai et al. (2016a)	Forward-inclusion and backward-elimination steps (stepwise covariate modeling)	Sex, Smoking status, Age, Height, Weight, BMI and the liver function index	Liver function index on CL/F BMI on V_P_/F	CL = 2540 (healthy) / 1,550 (mild) / 1,326 (moderate) mL/h × exp. (η_CL_) Q = 33,678 (healthy) / 38,800 (mild) / 63,554 (moderate) mL/h × exp. (η_Q_) V_P_ = 41,000 mL × [1 + 0.058 × (BMI - 27.00)] × exp. (η _VP_) V_C_ = 51,400 mL KAMAX = 0.86 h^−1^ RA = 0.653 × exp. (η_RA_) h^−1^ GAM1 = 4.57
3	Desai et al. (2016b)	Forward inclusion and backward elimination steps (stepwise covariate modeling)	Weight, BMI, Height, Lean body mass, Age, ALT, AST, TBILI, ALB, ALKPHOS, Race, Sex, SP and con-meds	Race on CL/F BMI, SP on V_P_/F	CL = 2.36 (Caucasian) / 1.51 (Asian) L/h × exp. (η_CL_) V_C_ = 49.1 L V_P_ = 417 × (1 + 0.060) × (BMI - 24.80) × exp. (η_VP_) Q = 26.6 L/h KAMAX = 1.08 h^−1^ RA = 0.72 × exp. (η_RA_) h^−1^ GAM1 = 4.88
4	Kovanda et al. (2017)	Covariate incorporation into secondary bioavailability equation using nonparametric population PK modeling	Presence of mucositis (yes/no)	NR	NR
5	Desai et al. (2019)	Forward inclusion and backward elimination step (stepwise covariate modeling)	Age, Weight and BMI	Weight on V_P_/F Age and Sex as grouping factors for CL/F	CL = 1.996 L/h (male) / 2.13 L/h (nonelderly female) / 1.44 L/h (elderly female) V_P_ = 263 × [1 + 0.021 × (WT - 72)] L (male), 263 × [1 + 0.443 × (WT - 72)] L (nonelderly female), 263 × [1 + 0.654 × (WT - 72)] L (elderly female) Q = 22.2 L/h KAMAX = 0.426 h^−1^ RA = 0.664 h^−1^ GAM1 = 4.75
6	Wu et al. (2020)	Forward inclusion and backward elimination steps (stepwise covariate modeling)	Sex, Age, Height, Weight, AIBW, BSA, BMI, SCR, CLCR, AST, ALP, ALB, TB, ALT, Types of transplant (lung, liver and others) and Recipients requiring renal replacement (HD, CRRT)	Sex on CL/F	CL = 4.28 (female) / 2.73 (male) L/h × exp. (η _CL_) V_P_ = [468 + 39.7 × (BMI - 25.38)] L × exp.(η_V2_) Q = 37.4 L/h
7	Arrieta et al. (2021)	Forward inclusion and backward elimination steps (stepwise covariate modeling)	Age, Sex, Race, BMI, Serum creatinine, ALT, AST, Total bilirubin, Albumin and AP	NR	NR
8	Cojutti et al. (2021)	Univariate analysis based on physiological plausibility and OFV	Age, Weight, BMI, bilirubin, liver enzymes, Coadministered CYP3A4 inhibitors/inducers	NA	NR
9	Jansen et al. (2023)	Univariate analysis based on physiological plausibility and OFV	Serum ALB, Total protein, urea, Total bilirubin, Age, Sex, TBW, Height, APACHE II and SOFA score	NR	C _unbound_ = [− k_d_- B _max_+ C_total_ + sqrt ((k_d_ + B_max_ − C_total_) (2)+ 4 × C_total_ × k_d_)]/2, with k_d_ fixed to 0.0806 mg/L
10	Shirae et al. (2023)	Forward inclusion and backward elimination steps (stepwise covariate modeling)	Weight, LBM, BMI, Height, CLCR, Age, Alanine aminotransferase, Aspartate aminotransferase, TB, ALB, Alkaline phosphatase, Sex, Subject’s condition, Concomitant use of CYP3A4 inhibitors, Concomitant use of CYP3A4 strong inhibitors, Concomitant use of CYP3A4 moderate/weak inhibitors and female >65 years old	LBM and CLCR on CL/F BMI and COND on V_P_/F COND on Q	CL = 1.74 × (LBM/49.19)^1.15^ × (CLCR/100.37)^-0.29^ L/h × exp. (η _CL_) V_C_ = 25.5 L V_P_ = 122 × (BMI/20.47)^2.25^ × (1 + 2.21 × COND) × exp.(η_V3_) Q = 19.2 × (1 + 10.59 × COND) L/h × exp. (η _Q_) KAMAX = 0.84 h^−1^ RA = 0.73 × exp. (η _RA_) h^−1^ GAM1 = 4.76 COND = 0 (healthy) / 1 (patient)
11	Perez et al. (2023)	LRT; Forward inclusion and backward elimination steps (stepwise covariate modeling)	Age, Weight, lean body weight, BMI, percentage body fat, AST, ALT, Total bilirubin, ALB, CKD-EPI, SOFA score, SAPS II, RRT and ECMO	RRT on CL/F	CL_i_ = 3.98 × e^(0.45 × RRT)^
12	Martín-Cerezuela et al. (2024)	Forward inclusion and backward elimination steps (stepwise covariate modeling)	Age, Sex, Weight, SAPS3, creatinine, Liver enzymes and extracorporeal devices (CRRT, ECMO)	NR	NR
13	Peña-Lorenzo et al. (2025)	Forward inclusion and backward elimination steps (stepwise covariate modeling)	Sex, Age, Height, TBW, BSA, BMI, ALB, Total proteins, Total bilirubin, Aspartate aminotransferase, Alanine aminotransaminase, Alkaline phosphatase, γ-glutamyl transferase, Urate, Creatinine, Estimated glomerular filtration rate, CRP, hemoglobin and Absolute neutrophil counts	BSA, ALB on CL/F	CL/*F* = 3.83 × (BSA/1.8)^1.8^ × (1–0.19 × (ALB-3.1))L/h V/*F* = 226 L(fixed) Ka = 22.6 h^−1^(fixed)
14	Rabault et al. (2025)	Conditional Sampling use for Stepwise Approach based on Correlation test (COSSAC)	Age, Weight, Sex, BMI, SOFA, ALB, Bilirubin, Creatinine, RRT, ECMO, Vasopressor use, CYP450 inducer/inhibitor co-medication, Liver function markers, Inflammatory markers, Organ support variables	NA	NR

TBW, total body weight; APACHE II, physiology and chronic health evaluation II; SOFA, sequential organ failure assessment; B _max_, maximum binding capacity; BSA, body surface area; BMI, body mass index; ALB, albumin; CL/F, apparent clearance; V/F, apparent volume of distribution; K_a_, first-order absorption rate constant; k _d_, the equilibrium dissociation constant; C _unbound_, the protein unbound isavuconazole concentration; C _total,_ the total isavuconazole concentration; CL, clearance; V_P_, volume of distribution of peripheral compartment; Q, intercompartmental clearance; eGFR, Estimated glomerular filtration rate; ALT, alanine transaminase; AST, aspartate aminotransferase; TBILI, total bilirubin concentration; ALKPHOS, alkaline phosphate; SP, subjects and patients; AIBW, adjusted ideal body weight; ALP, alkaline phosphatase; TB, total bilirubin; SCR, serum creatinine; CLCR, creatinine clearance; HD, hemodialysis; CRRT, continuous renal replacement therapy; CRP, C-reactive protein; VC, central compartment volume; LBM, lean body mass; LRT, likelihood ratio test; SAPS II, simplified acute physiology score II; RRT, renal replacement therapy; ECMO, extracorporeal membrane oxygenation; V_P_, peripheral compartment volume; AP, alkaline phosphatase; SAPS3, simplified acute physiologic score; NA, not applicable; NR, No Record.

The identified covariates affecting CL and V are presented as forest plots in Figure 3. Overall, most covariates exerted only modest effects on isavuconazole clearance and volume of distribution. Compared with CL, V exhibited greater variability in response to covariate effects. Body weight and BMI were the most frequently identified determinants of V and consistently showed a positive association with an increased volume of distribution. In addition, differences in disease status between healthy subjects and patients had a more pronounced impact on V, with several studies reporting effect ranges that exceeded those observed for most other covariates. In contrast, the effects of body weight, ALB concentration, creatinine clearance, hepatic function markers, and race on CL were generally limited. These findings suggest that the pharmacokinetic parameters of isavuconazole are relatively robust, with identified covariates contributing only modestly to interindividual variability.

**Figure 3:**
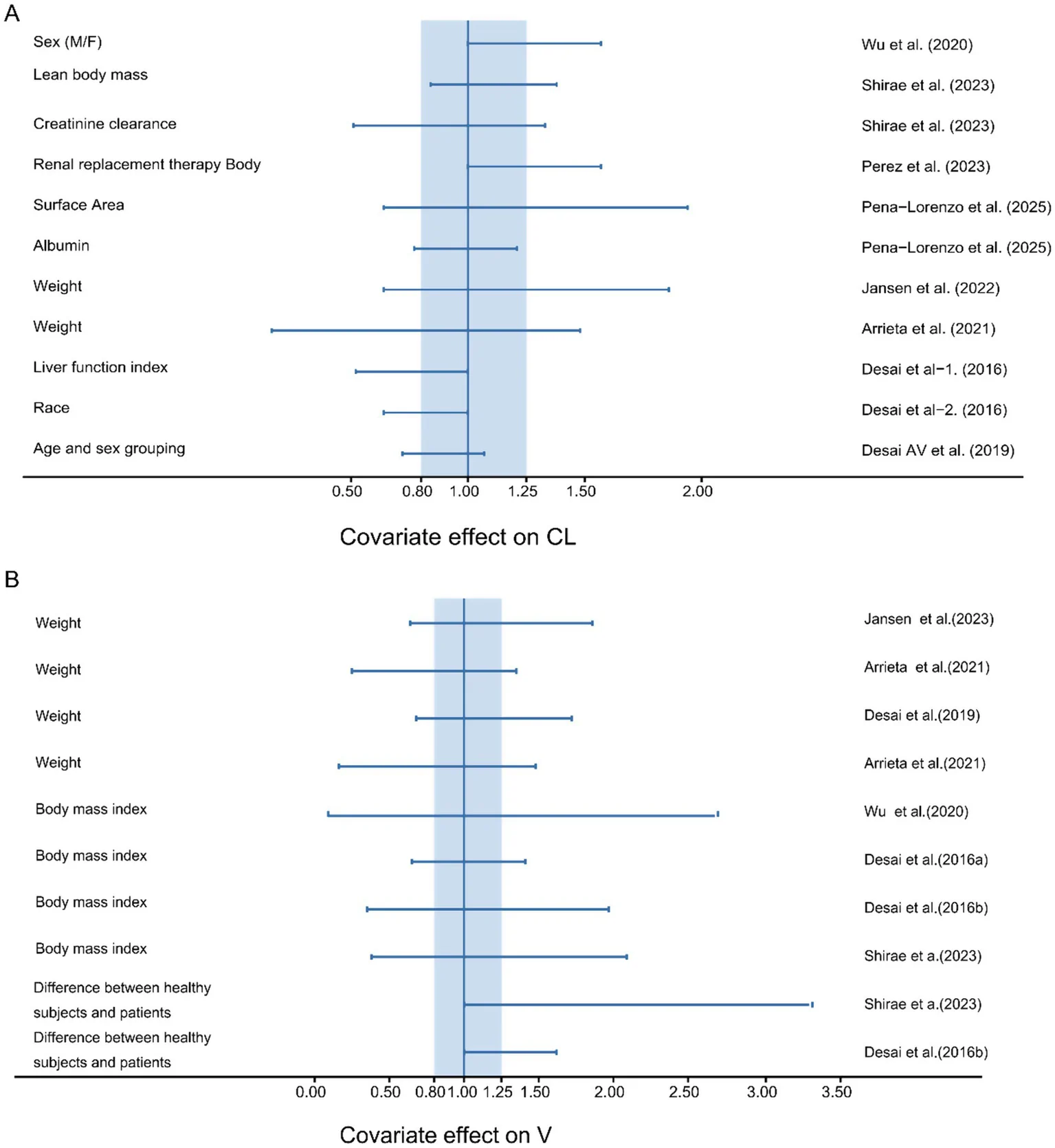
Covariate effect on pharmacokinetic parameters of isavuconazole. **(A)** The effect of covariates on the CL (L/h) of isavuconazole in the included studies. **(B)** The effect of covariates on V (L) of isavuconazole in included studies. The horizontal bars represent the covariate effect on clearance in each study. The typical value of clearance in each study was considered to be 1. The effect of each covariate for clearance is displayed by the ratio of clearance in the range of each covariate to the typical clearance value. The shaded area ranges from 0.8 to 1.25.

### Model library application

The simulated PTAs at fixed maintenance doses were assessed, and the results are shown in Figure 4. For a susceptive threshold of MIC = 1 mg/L, the PTA trajectories across different populations exhibit a distinct divergence. The simulated target achievement of three models fell below 90% (Peña-Lorenzo et al., 2025; Perez et al., 2023; Rabault et al., 2025). In contrast, the remaining models maintained relatively good target achievement rates at an MIC of 1 mg/L, including the only model specifically developed for pediatric patients (Arrieta et al., 2021).

**Figure 4:**
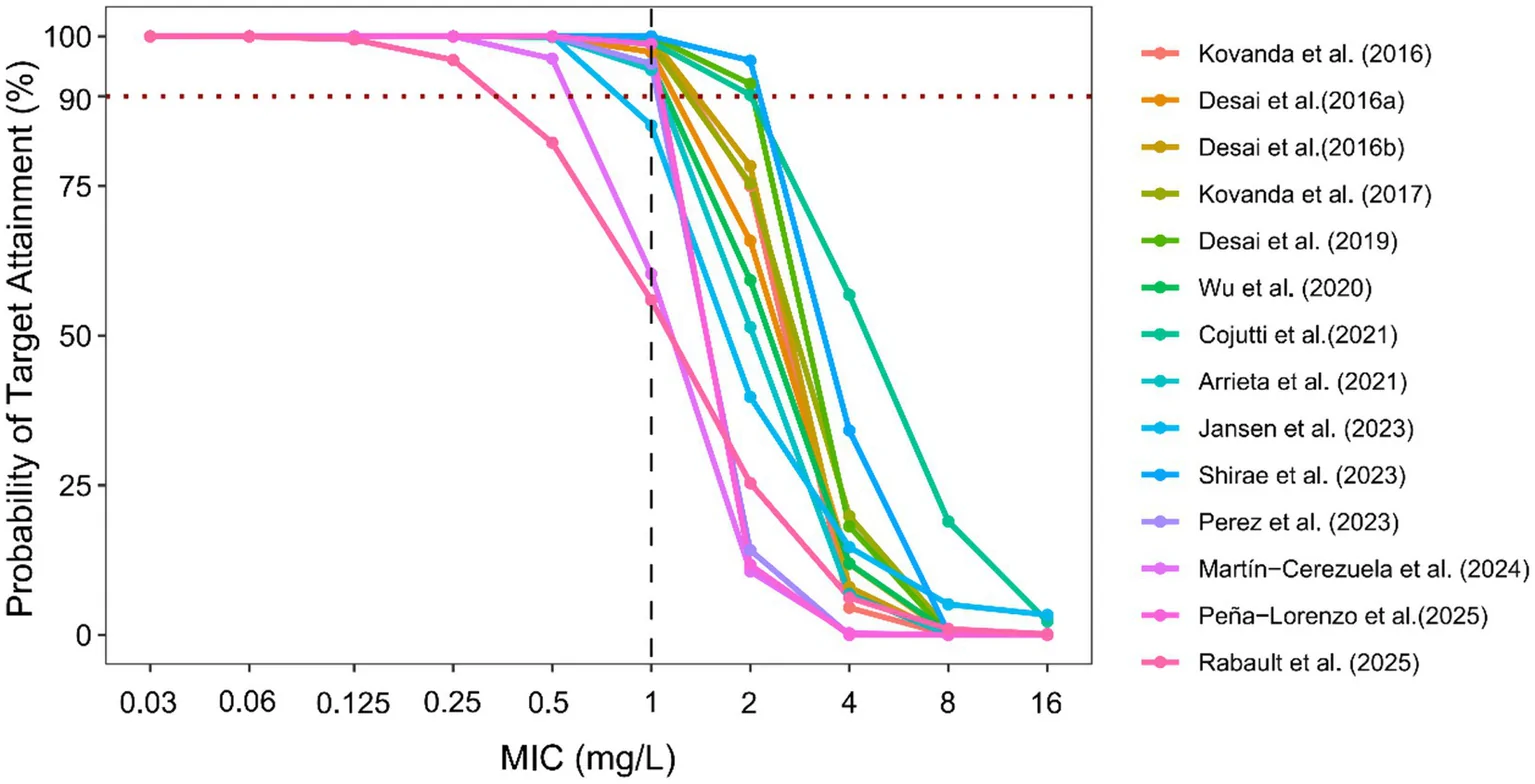
The probability target achievement of isavuconazole for each model. The red dashed line represents a 90% target attainment rate, while the vertical dashed line indicates the reference MIC value of 1 mg/L.

## Discussion

Based on a systematic synthesis of previous PPK studies, this research developed a standardized isavuconazole population pharmacokinetic model library. Through model reproduction and simulation, covariate effect evaluation, and pharmacodynamic analysis, these models were comprehensively compared and assessed. This library not only provides a foundational framework for subsequent external validation and model updates but also offers a reusable platform for MIPD and dose optimization studies. The library would benefit further research on precision isavuconazole dosing.

The 14 included models had high reporting quality and covered a diversity of patients. However, some studies, especially postmarketing PPK studies, had limited sample sizes. The PK of isavuconazole in specific patient populations needs more data. Overall, most studies reported typical clearance values within the range of 1.33–5.34 L/h. However, compared with healthy subjects, the typical clearance decreased by approximately 39% and 48% in patients with mild and moderate hepatic impairment in Desai et al. (2016b) respectively, whereas clearance increased by approximately 57% in patients receiving continuous renal replacement therapy in Perez et al. (2023). These findings suggest that disease-related factors may contribute more substantially to the pharmacokinetic variability of isavuconazole than demographic characteristics. Nevertheless, as the current evidence is largely derived from individual studies, further investigations are warranted to confirm the generalizability and clinical relevance of these observations.

To date, no covariates have been identified that have been repeatedly validated across different studies and have significant clinical implications. But the absence of reproducible covariates should not be interpreted as evidence that these factors are clinically irrelevant. Instead, it should be regarded as an indication that their effects may depend on specific circumstances. Such effects need to be evaluated through standardized covariates and externally validated in larger and more clearly defined populations. Currently reported isavuconazole pharmacokinetic variability is highly population dependent, that is, the effects of covariates observed in different studies may only partially reflect the pathophysiological characteristics of specific populations but may not be universally applicable to studies involving all patients. Nevertheless, several noteworthy patterns emerged from the comparison of covariate effects across studies. First, body size–related variables such as BMI and body weight were incorporated into multiple final models, although their primary influence was on peripheral volume of distribution rather than clearance. This finding is consistent with the physicochemical characteristics of isavuconazole, including its large apparent volume of distribution and high lipophilicity (Chen et al., 2023; Lewis et al., 2022), suggesting that body size may predominantly influence tissue distribution rather than drug elimination.

In contrast, covariates associated with clearance were more frequently related to disease status than to demographic characteristics. These included hepatic function, creatinine clearance, continuous renal replacement therapy (RRT), albumin (ALB) concentration, and disease condition (healthy subjects versus patients). Compared to previous reviews, this study included models validated at lower protein levels that lead to increased CL and incorporated ALB into the covariate model for CL. This partially addresses the research gap regarding the impact of ALB concentration on isavuconazole exposure (Chen et al., 2023; Peña-Lorenzo et al., 2025). Given that isavuconazole is primarily metabolized through hepatic pathways, the reduction in clearance observed in patients with hepatic impairment is biologically plausible (Tan et al., 2025; Desai et al., 2016b). Furthermore, critical illness–related factors such as hypoalbuminemia, renal replacement therapy, and severe disease states may indirectly affect drug exposure through alterations in protein binding, tissue perfusion, and organ function (Tan et al., 2025).

This study conducted simulations of the included models to further evaluate the pharmacodynamic target attainment under the standard dosing regimen. In our analysis, PTA values below 90% were primarily observed in special patient populations, including patients with hematological diseases, critically ill patients, and patients with abnormal covariate subgroups. In the model by Peña-Lorenzo et al. (2025), BSA and serum albumin were identified as significant determinants of clearance, potentially leading to insufficient drug exposure in a subset of patients. Similarly, Rabault et al. (2025) reported that critically ill patients were at an increased risk of achieving suboptimal isavuconazole exposure under the standard dosing regimen. Perez et al. (2023) conducted a study on CAPA intensive care patients, first suggesting that in this specific population, an intensified loading dose and maintenance dosing strategy may be needed, combined with TDM for individualized dose adjustment. This is consistent with the findings from the boxplots in this study. As shown in the boxplots, the AUC distributions are relatively concentrated, with the medians of most studies falling within a similar range; however, some studies still exhibit a wide range of individual exposures, suggesting a risk of under- or over-exposure in specific patient subgroups.

Although some experts considered that isavuconazole exhibited low pharmacokinetic variability and routine TDM may not be needed, our findings suggest that patients with special conditions may be at risk of suboptimal exposure. Most covariates had a clinically significant influence on isavuconazole PK. Moreover, the inconsistency in covariate screening among models indicated the uncertainty of extrapolating the performance of models. Therefore, the potential utility of MIPD may be greater in selected high-risk populations than in the broader population of patients receiving isavuconazole. Further research is warranted to establish whether MIPD can improve drug exposure and translate into meaningful clinical benefits.

Our research has certain limitations. Since most of the study participants included in our analysis were adults, we were unable to comprehensively investigate and compare the pharmacokinetics in children and infants. Furthermore, the significant heterogeneity among the included studies led to an inability to cross-validate results. Although the included studies underwent internal evaluation, none underwent external evaluation, which is considered the most rigorous form of assessment. Only a low limit to achieve the PK/PD target was considered when assessing dosing adequacy, and the high limit to reduce toxicity was not considered. Finally, although C_max_ is influenced not only by compartmental structure but also by absorption characteristics, route of administration, infusion duration, and sampling design, it may provide additional insights into distribution characteristics and early exposure profiles when evaluated under standardized dosing and simulation conditions. Therefore, further comparative studies with harmonized assumptions and simulation settings are warranted to better characterize differences in peak exposure among pharmacokinetic models.

## Conclusion

This study successfully developed a population pharmacokinetic library for isavuconazole, which supports its MIPD. The current dosing regimen of isavuconazole is sufficient for common circumstances and does not require adjustment. However, specific patient groups, such as critically ill patients, hematological patients and patients with abnormal covariates, for whom dose adjustment should be prioritized.

## Data Availability

The original contributions presented in the study are included in the article/Supplementary material, further inquiries can be directed to the corresponding authors.
