# Supplementary material for: Population pharmacokinetic model library for isavuconazole: development and quality control
Source: Front Microbiol. 2026 Oct 6;17:1930025. doi: 10.3389/fmicb.2026.1930025 (PMC13680455; doi:10.3389/fmicb.2026.1930025)
Supplement: Supplementary file 1 [file Data_Sheet_1.DOCX]

Table S1. Check List for quality assessment of included studies

| **Section** | **Item** |
| --- | --- |
| Title & Abstract | Study objective clearly stated  Isavuconazole clearly identified  Population pharmacokinetic analysis mentioned  Key methods summarized  Key findings summarized |
| Introduction | Scientific background provided  Study objective clearly defined |
| Methods | Study design described  Study population reported  Inclusion/exclusion criteria reported  Sample size reported  Dosing regimen described  Sampling schedule reported  Bioanalytical method described  Assay validation reported  Structural model candidates described  Absorption model described  Interindividual variability model described  Residual error model described  Covariates screened reported  Covariate selection strategy described  Concomitant medications and potential drug interactions reported  Software reported  Estimation algorithm reported  Internal validation method described  External validation method described (if applicable)  Simulation method described (if applicable) |
| Results | Number of subjects reported  Number of observations reported  Missing or excluded observations reported  Demographic characteristics reported  Clinical characteristics reported  Final model structure reported  Parameter estimates reported  Parameter precision reported  Interindividual variability estimates reported  Residual variability reported  Final model evaluation plots reported  Significant covariates identified  Covariate effects quantified  Simulation outcomes reported (PTA/AUC/C_ss_/CFR) |
| Discussion & Conclusion | Main findings summarized  Clinical implications discussed  Generalizability or clinical applicability discussed  Model limitations discussed  Conclusions supported by results |

TableS2. Quality assessment scores of the included PPK studies

| Models | 1 | 2 | 3 | 4 | 5 | 6 | 7 | 8 | 9 | 10 | 11 | 12 | 13 | 14 |
| --- | --- | --- | --- | --- | --- | --- | --- | --- | --- | --- | --- | --- | --- | --- |
| Total scores | 44.0 | 41.0 | 42.5 | 44.0 | 38.5 | 43.0 | 39.5 | 45.0 | 45.0 | 42.0 | 44.0 | 45.0 | 34.5 | 44.0 |
| Study objective clearly stated | 1 | 1 | 1 | 1 | 1 | 1 | 1 | 1 | 1 | 1 | 1 | 1 | 1 | 1 |
| Isavuconazole clearly identified | 1 | 1 | 1 | 1 | 1 | 1 | 1 | 1 | 1 | 1 | 1 | 1 | 1 | 1 |
| Population pharmacokinetic analysis mentioned | 1 | 1 | 1 | 1 | 1 | 1 | 1 | 1 | 1 | 1 | 1 | 1 | 1 | 1 |
| Key methods summarized | 1 | 1 | 1 | 1 | 1 | 1 | 1 | 1 | 1 | 1 | 1 | 1 | 1 | 1 |
| Key findings summarized | 1 | 1 | 1 | 1 | 1 | 1 | 1 | 1 | 1 | 1 | 1 | 1 | 1 | 1 |
| Scientific background provided | 1 | 1 | 1 | 1 | 1 | 1 | 1 | 1 | 1 | 1 | 1 | 1 | 1 | 1 |
| Study objective clearly defined | 1 | 1 | 1 | 1 | 1 | 1 | 1 | 1 | 1 | 1 | 1 | 1 | 1 | 1 |
| Study design described | 1 | 1 | 1 | 1 | 1 | 1 | 1 | 1 | 1 | 1 | 1 | 1 | 1 | 1 |
| Study population reported | 1 | 1 | 1 | 1 | 1 | 1 | 1 | 1 | 1 | 1 | 1 | 1 | 1 | 1 |
| Inclusion/exclusion criteria reported | 1 | 1 | 0.5 | 1 | 1 | 1 | 1 | 1 | 1 | 0.5 | 1 | 1 | 1 | 1 |
| Sample size reported | 1 | 1 | 1 | 1 | 1 | 1 | 1 | 1 | 1 | 1 | 1 | 1 | 1 | 1 |
| Dosing regimen described | 1 | 1 | 1 | 1 | 1 | 1 | 1 | 1 | 1 | 1 | 1 | 1 | 1 | 1 |
| Sampling schedule reported | 1 | 1 | 1 | 1 | 1 | 1 | 1 | 1 | 1 | 1 | 1 | 1 | 1 | 1 |
| Bioanalytical method described | 1 | 1 | 0 | 1 | 1 | 1 | 1 | 1 | 1 | 0 | 1 | 1 | 1 | 1 |
| Assay validation reported | 1 | 1 | 0 | 1 | 0 | 1 | 1 | 1 | 1 | 0 | 1 | 1 | 1 | 1 |
| Structural model candidates described | 1 | 1 | 1 | 1 | 1 | 1 | 1 | 1 | 1 | 1 | 1 | 1 | 0 | 1 |
| Absorption model described | 1 | 1 | 1 | 1 | 1 | 0 | 1 | 1 | 1 | 1 | 0 | 1 | 0 | 0 |
| IIV model described | 1 | 0 | 1 | 1 | 1 | 1 | 0 | 1 | 1 | 1 | 1 | 1 | 0 | 1 |
| Residual error model described | 1 | 0 | 1 | 1 | 1 | 1 | 0 | 1 | 1 | 0.5 | 1 | 1 | 0 | 1 |
| Covariates screened reported | 1 | 1 | 1 | 1 | 1 | 1 | 1 | 1 | 1 | 1 | 1 | 1 | 1 | 1 |
| Covariate selection strategy described | 1 | 1 | 1 | 1 | 1 | 1 | 1 | 1 | 1 | 1 | 1 | 1 | 1 | 1 |
| Concomitant medications reported | 0.5 | 1 | 1 | 0.5 | 0 | 1 | 1 | 1 | 1 | 1 | 1 | 1 | 0.5 | 1 |
| Software reported | 1 | 1 | 1 | 1 | 1 | 1 | 1 | 1 | 1 | 1 | 1 | 1 | 1 | 1 |
| Estimation algorithm reported | 1 | 1 | 1 | 1 | 1 | 1 | 0 | 1 | 1 | 1 | 1 | 1 | 1 | 1 |
| Internal validation method described | 1 | 1 | 1 | 1 | 1 | 1 | 1 | 1 | 1 | 1 | 1 | 1 | 0 | 1 |
| External validation method described | 0 | 0 | 0 | 0 | 0 | 0 | 0 | 0 | 0 | 0 | 0 | 0 | 0 | 0 |
| Simulation method described | 1 | 1 | 1 | 1 | 0 | 1 | 1 | 1 | 1 | 1 | 1 | 1 | 0 | 1 |
| Number of subjects reported | 1 | 1 | 1 | 1 | 1 | 1 | 1 | 1 | 1 | 1 | 1 | 1 | 1 | 1 |
| Number of observations reported | 1 | 1 | 1 | 1 | 1 | 1 | 1 | 1 | 1 | 1 | 1 | 1 | 1 | 1 |
| Missing/excluded observations reported | 1 | 0 | 1 | 1 | 1 | 0 | 1 | 1 | 1 | 1 | 1 | 1 | 0 | 1 |
| Demographic characteristics reported | 1 | 1 | 1 | 1 | 1 | 1 | 1 | 1 | 1 | 1 | 1 | 1 | 1 | 1 |
| Clinical characteristics reported | 1 | 1 | 1 | 1 | 1 | 1 | 1 | 1 | 1 | 1 | 1 | 1 | 1 | 1 |
| Final model structure reported | 1 | 1 | 1 | 1 | 1 | 1 | 1 | 1 | 1 | 1 | 1 | 1 | 1 | 1 |
| Parameter estimates reported | 1 | 1 | 1 | 1 | 1 | 1 | 0.5 | 1 | 1 | 1 | 1 | 1 | 1 | 1 |
| Parameter precision reported | 0.5 | 1 | 1 | 0.5 | 1 | 1 | 0.5 | 1 | 1 | 1 | 1 | 1 | 1 | 1 |
| IIV estimates reported | 1 | 1 | 1 | 1 | 1 | 1 | 0.5 | 1 | 1 | 1 | 1 | 1 | 1 | 1 |
| Residual variability reported | 1 | 1 | 1 | 1 | 1 | 1 | 0.5 | 1 | 1 | 1 | 1 | 1 | 0 | 1 |
| Final model evaluation plots reported | 1 | 1 | 1 | 1 | 0.5 | 1 | 0.5 | 1 | 1 | 1 | 1 | 1 | 0 | 1 |
| Significant covariates identified | 1 | 1 | 1 | 1 | 1 | 1 | 1 | 1 | 1 | 1 | 1 | 1 | 1 | 1 |
| Covariate effects quantified | 1 | 1 | 1 | 1 | 1 | 1 | 1 | 1 | 1 | 1 | 1 | 1 | 1 | 1 |
| Simulation outcomes reported | 1 | 1 | 1 | 1 | 0 | 1 | 1 | 1 | 1 | 1 | 1 | 1 | 0 | 1 |
| Main findings summarized | 1 | 1 | 1 | 1 | 1 | 1 | 1 | 1 | 1 | 1 | 1 | 1 | 1 | 1 |
| Clinical implications discussed | 1 | 1 | 1 | 1 | 1 | 1 | 1 | 1 | 1 | 1 | 1 | 1 | 1 | 1 |
| Applicability discussed | 1 | 1 | 1 | 1 | 1 | 1 | 1 | 1 | 1 | 1 | 1 | 1 | 1 | 1 |
| Model limitations discussed | 1 | 1 | 1 | 1 | 1 | 1 | 1 | 1 | 1 | 1 | 1 | 1 | 1 | 1 |
| Conclusions supported by results | 1 | 1 | 1 | 1 | 1 | 1 | 1 | 1 | 1 | 1 | 1 | 1 | 1 | 1 |

The models are: 1, Kovanda (2016) ;2, Desai (2016a) ;3, Desai (2016b) ;4, Kovanda (2017);5, Desai, AV (2019) ;6, Wu et al. (2020); 7, Arrieta (2021) ;8, Cojutti (2021) ;9, Jansen (2023);10, Shirae (2023);11, Perez (2023) ;12, Peña-Lorenzo (2025) ;13, Martín-Cerezuela (2024) ;14, Rabault (2025)

Table S3. Experimental designs and dosing regimens of the included studies.

| **ID** | **Author** | **Daily Dose** | **Sampling Schedule** | **Bioassay** |
| --- | --- | --- | --- | --- |
| 1 | Kovanda et al. | A loading dose of 372 mg isavuconazonium sulfate (equivalent to 200 mg isavuconazole) q8h for six doses (i.v. or p.o.), followed by a maintenance dose of 200 mg q24h. | On days 7,14, 42 and at the end of of treatment, samples collected at 24 h after the start of the infusion or the p.o. dose  the previous day (i.e., trough concentration). | LC-MS/MS |
| 2 | Desai A, et al. | A single dose of isavuconazonium sulfate at 186 mg (equivalent to 100 mg of ISA), either p.o. or i.v. (The infusion time was 2h). | Prior to administration and at 0.5, 1, 2, 3, 4, 6, 8, 10, 24, 48, 72, 96, 120, 144, 168, 216, 288, 360, 432, and 480 h after administration of the drug | NR |
| 3 | Desai A, et al. | 372 mg isavuconazonium sulfate i.v. tid for 6 doses (i.e., days 1 and 2), followed by 372 mg isavuconazonium sulfate administered qd, either p.o. or i.v., from day 3 to the end of treatment. | Predose-840h | NR |
| 4 | Kovanda et al. | A loading dose of 372 mg isavuconazonium sulfate (equivalent to 200 mg isavuconazole) q8h for six doses (i.v. or p.o.), followed by a maintenance dose of 200 mg q24h up to 84 or 180 days depending on trial. | On days 7, 14, 42, and end of therapy (EOT); trough sampling targeted at 24 h post-dose; full 24-h PK profiles in subset of 43 patients | LC-MS/MS |
| 5 | Desai AV, et al. | 372 mg isavuconazonium sulfate orally | At 0.5, 1, 1.5, 2, 3, 4, 6, 8, 10, 12, 16, 20, 24, 36, 48, 72, 120, 168, 228, 240, and 336 h postdose. | NR |
| 6 | Wu et al. | 372 mg isavuconazonium sulfate tid, followed by 372 mg qd. (i.v.) | Prior to (0 h) and at 1, 2, 4, 6, 8, 12, 16 and 24 h following administration of a minimum of 7 doses of ISA (26 recipient)  Randomly during prophylaxis with ISA (with ISA53 recipients) | UPLC-MS/MS |
| 7 | Arrieta et al. | 10 mg/kg body weight (maximum, 372 mg) isavuconazonium sulfate either i.v. or p.o. | i.v. cohort: on days 3, 7, and 28 or EOT visit (within 15min prior to the start of the i.v. infusion, within 5min of infusion completion, and within 4 to 8, 8 to 12, and 16 to 24 h after the start of infusion) oral cohort: on day 7 (±1 day), with samples taken predose, and at 1,2, 3, 4 (±10 min), 6 (±30 min), 8 (±30 min), and 24 h after study drug administration; the 24 h sample was taken ≤1 h before the next dose | LC-MS/MS |
| 8 | Cojutti et al. | A loading dose of 200 mg ISA q8h for six doses, followed by a maintenance dose of 200 mg q24h.(p.o.) | TDM samples collected at steady state as trough concentrations (C_trough_); | LC-MS/MS |
| 9 | Jansen et al. | A loading dose of 200 mg ISA q8h for six doses, followed by a maintenance dose of 200 mg q24h.(i.v.) | On days 3-7 of treatment, samples collected at t = 0 (pre-dose), and t = 0.5, 1, 2, 4, 6, 8, and 12 h post-infusion Optional sample 96–300 h after discontinuation | LC-MS/MS |
| 10 | Shirae et al. | A loading dose of 200 mg ISA q8h for six doses, followed by a maintenance dose of 200 mg q24h. | NR | NR |
| 11 | Perez et al. | Six (33.3%) patients: prolonged loading dose for 72 h, and the mean maintenance dose was 264 mg/day.  The other :200mg ISA tid for 6 doses, followed by 200mg qd.(i.v.) | Trough concentrations, collected during routine TDM median time to first sampling was 5 days (IQR: 4.25–7.5) after therapy initiation | HPLC |
| 12 | Martín-Cerezuela et al. | A loading dose of 200 mg ISA q8h for six doses, followed by a maintenance dose of 200 mg q24h. (The infusion time was 60 min) | At predose, 1 h and 12 h after the last dose | HPLC |
| 13 | Peña-Lorenzo et al. | A loading dose of 200 mg ISA q8h for six doses, followed by a maintenance dose of 200 mg q24h, administered 12–24 h after the last loading dose. (i.v.) | At 0.5, 2, and 24 h following intravenous administration, or at 2, 3.5, and 24 h after oral dosing. | Reversephase UPLC |
| 14 | Rabault et al. | A loading dose of 200 mg ISA q8h for six doses, followed by a maintenance dose of 200 mg q24h | Routine TDM without structured sampling schedule after at least 2 treatment days；  46 trough concentrations collected | Reversephase UPLC-MS/MS |
|  |  |  |  |  |

Abbreviations: LC‒MS/MS, liquid chromatography‒tandem mass spectrometry; UPLC, ultra-performance liquid chromatography; UPLC-MS/MS, ultra-performance

liquid chromatography-tandem mass spectrometry assay; ISA, isavuconazole; TDM, therapeutic drug monitoring; EOT, end-of-treatment; C_trough_, trough concentration;

C_max_, peak plasma concentration; HPLC, high-performance liquid chromatography; NR, no record.
